# Silibinin-Loaded Proniosomal Gel for Cutaneous Application: Pharmaco-Technical Characterization and In Vitro–In Ovo Biocompatibility

**DOI:** 10.3390/gels12060504

**Published:** 2026-06-05

**Authors:** Andreea Smeu, Ioana Olariu, Iasmina Marcovici, Diana Haj-Ali, Lavinia Vlaia, Vicențiu Vlaia, Alina Tănase, Raluca Mioara Cosoroabă, Vlad Socoliuc, Cristina Adriana Dehelean

**Affiliations:** 1 Doctoral School, “Victor Babes” University of Medicine and Pharmacy, Eftimie Murgu Square No. 2, 300041 Timisoara, Romania; andreea.smeu@umft.ro (A.S.); diana.haj-ali@umft.ro (D.H.-A.); 2 Research Center for Pharmaco-Toxicological Evaluation, Faculty of Pharmacy, “Victor Babes” University of Medicine and Pharmacy, Eftimie Murgu Square No. 2, 300041 Timisoara, Romania; cadehelean@umft.ro; 3 University Clinic of Toxicology, Drug Industry, Management, Legislation, and Dermatopharmacy, Faculty of Pharmacy, “Victor Babes” University of Medicine and Pharmacy Timisoara, Eftimie Murgu Square No. 2, 300041 Timisoara, Romania; 4 Department II—Pharmaceutical Technology, Formulation and Technology of Drugs Research Center, “Victor Babes” University of Medicine and Pharmacy, Eftimie Murgu Square No. 2, 300041 Timisoara, Romania; olariu.ioana@umft.ro (I.O.); vlaia.lavinia@umft.ro (L.V.); 5 Department II—Organic Chemistry, Formulation and Technology of Drugs Research Center, “Victor Babes” University of Medicine and Pharmacy, Eftimie Murgu Square No. 2, 300041 Timisoara, Romania; vlaiav@umft.ro; 6 Faculty of Dental Medicine, “Victor Babes” University of Medicine and Pharmacy, Eftimie Murgu Square No. 2, 300041 Timisoara, Romania; tanase.alina@umft.ro (A.T.); cosoroaba.raluca@umft.ro (R.M.C.); 7 Laboratory of Magnetic Fluids, Center of Fundamental and Advanced Technical Research, Romanian Academy–Timisoara Branch, 24 Mihai Viteazul Avenue, 300223 Timisoara, Romania; vsocoliuc@gmail.com

**Keywords:** silibinin, proniosomal gel, pharmaco-technical evaluation, safety profile, keratinocytes, spheroids, reconstructed human tissue, irritant potential

## Abstract

The skin serves as the first line of defense, being highly prone to external damage. Silibinin (SIL) exerts skin-protective properties, but its topical use requires a suitable delivery system. Despite the growing interest in proniosomal platforms loaded with natural products, their application for the cutaneous delivery of SIL remains scarcely explored. This study proposes the pharmaco-technical characterization and preclinical safety evaluation of a SIL-loaded proniosomal gel (SIL-PG) for skin application. SIL-PG was produced using the coacervation phase separation technique, analyzed in terms of physicochemical and technological properties, and evaluated in vitro and in ovo for potential cytotoxic and irritant effects. SIL-PG retained a yellowish, creamy aspect, proper rheological behavior and spreadability, gradual in vitro drug release, sustained permeation, and an adequate safety profile, evidenced by the lack of cytotoxicity in HaCaT keratinocytes and spheroids and the absence of irritant potential in 3D EpiDerm™ reconstructed human tissues and on the chorioallantoic membrane. Overall, these findings emphasize SIL-PG as a potential pharmaceutical formulation for dermal use, with favorable pharmaco-technical characteristics and in vitro–in ovo biocompatibility.

## 1. Introduction

The skin constitutes the largest organ of the body, serving as the first line of defense against environmental insults (e.g., physical, chemical, or biological), while also preventing water loss [1,2]. Due to its constant exposure to external factors, the skin is highly prone to the toxic effects caused by oxidants, ultraviolet (UV) rays, and pollutants (i.e., cigarette smoke, heavy metals, and ozone) that trigger reactive oxygen species (ROS) formation or directly alter the cutaneous barrier function, leading to less or more severe dermatological conditions (e.g., edema, hyperplasia, sunburn, aging, and carcinogenesis, to name a few), when excessive exposure to such stressors surpasses the skin’s innate protective capability [3]. The growing interest in efficient products for the treatment of such disorders or for skincare applications has significantly increased the popularity of natural agents owing to their few side effects and diverse biological activities, such as antioxidant, anti-aging, moisturizing, rejuvenating, anti-inflammatory, antimicrobial, photoprotective, wound-healing, etc., properties [4,5,6,7,8].

One such herbal component is silibinin (SIL), a major flavonolignan constituent of silymarin extracted from the milk thistle plant (*Silybum marianum* L.), traditionally used in the treatment of cirrhosis and hepatitis. However, the therapeutic effects of SIL exceed its conventional recommendations, encompassing anti-inflammatory, anticancer, antiviral, antibacterial, and antioxidant properties [9,10]. Increasing evidence suggests that SIL presents innate properties that might redirect its use towards cutaneous applications. For instance, it has been established that this compound has protective capabilities against external stressors, reducing UV-induced apoptosis in skin cells and epidermal damage in mice [11,12], preventing fibrotic changes in the skin [13], and protecting keratinocytes from sunburn [12]. One study showed that SIL can prevent skin cancer incidence, promotion, and progression caused by chemical carcinogens or UV radiation [14], while others demonstrated its anti-tumor activity against basal cell carcinoma [15] and melanoma [16]. These skin-beneficial effects have further encouraged the development of topical products containing SIL as an active ingredient. Hence, a previous study demonstrated the in vivo wound-healing activity of SIL following incorporation into a hydrogel [17]. Additionally, other studies have also described the formulation and in vivo biological activity of SIL-loaded hydrogels that showed protective effects against UVB-induced skin damage by reducing edema and inflammatory responses [18], or by preventing ROS production, hyperkeratosis, acanthosis, and dermal infiltration of neutrophils [19]. Collectively, these promising results highlight the relevance of SIL-based skin formulations that constitute a highly attractive avenue to explore.

Among many available topical carriers, proniosomes are one of the most abundantly used vesicular platforms for transdermal drug delivery, being instilled with favorable features, including chemical and physical stability, advantageous transport, distribution, and storage, proper entrapment capacity, and enhanced skin permeability. In addition to these benefits, proniosomes present high versatility as they are able to deliver both hydrophobic and hydrophilic molecules, while facilitating depot release to the stratum corneum (SC), reducing administration frequency, boosting therapeutic efficacy, and ameliorating side effects [20,21,22]. These advantages were further translated into recently developed proniosomal gels loaded with botanical constituents (i.e., puerarin and curcumin) that showed promising therapeutic potential [21,23]. Still, there is an under-investigated area in the existing literature regarding the application of proniosomal platforms for the cutaneous delivery of SIL.

The use of dermal preparations for therapeutic, cosmetic, or other purposes has faced a significant increase globally, and since many are available as over-the-counter products, collecting enough data to ensure their safety is essential [24]. In fact, among the most common concerns (e.g., aspect, delivery, efficacy, etc.) regarding the application of topical formulations, skin tolerability stands as an absolute priority and should be rigorously assessed to ascertain consumer safety before product use [25,26]. Such toxicological studies can be efficiently performed using preclinical models [24]. Notably, driven by stricter regulations and growing interest in the development of alternative, ethical, and refined testing methods, there is an accelerated transition from animal-based safety evaluation of cosmetics and pharmaceuticals towards new approach methodologies (NAMs) [27,28].

In this context, the present study aimed to conduct a pharmaco-technical evaluation and multi-model, non-animal safety screening of a SIL-loaded proniosomal gel (SIL-PG) for potential dermal application. The research initially evaluated the physicochemical characteristics, rheological behavior, spreadability, in vitro drug release, and permeability of SIL-PG. In parallel, the skin biocompatibility of the formulation was comparatively assessed against SIL and the blank gel base using (two-dimensional) 2D and (three-dimensional) 3D in vitro models, while the potential irritant effect and vascular toxicity were assessed in ovo. This integrated approach, combining functional characterization with safety assessment in complementary preclinical models, provided a broader understanding of the pharmaceutical features and biocompatibility of SIL-PG as a potential formulation candidate for cutaneous utilization.

## 2. Results and Discussion

### 2.1. Formulation and Characterization

SIL is a plant-derived flavolignan instilled with broad-spectrum bioactivity, including skin protective properties [29,30]. Proniosomal systems present multiple benefits as topical formulations, becoming a highly attractive strategy for the delivery of natural compounds [21,23]. However, at present, the development of SIL-containing proniosomal gels remains insufficiently studied. We have previously demonstrated the encapsulation of the natural flavonoid rutin into a proniosomal gel and the high in vitro biocompatibility of the obtained formulation (containing 0.3% bioactive ingredient) using the EpiDerm™ microtissues [31]. Built upon our previous findings, the main objective of the present research was the pharmaco-technical assessment and safety validation of SIL-PG across multiple preclinical models relevant for skin toxicity studies, including HaCaT keratinocytes, HaCaT spheroids, EpiDerm™ tissues, and the chorioallantoic membrane (CAM) of fertilized chicken eggs. The formulation study was exploratory rather than optimization-driven; therefore, the aim of the formulation stage was to develop a carrier system suitable for the encapsulation of SIL.

Blank proniosomal gel (BPG) and SIL-PG were prepared in fresh batches using the method and formulation components described in our previous work and further characterized. To obtain stable proniosomal gel formulations intended for cutaneous application, the following key excipients are used: (a) non-ionic surfactants acting as vesicle forming agents, with wetting and emulsifying effects, therefore improving the solubility and permeability of the encapsulated active ingredient; (b) cholesterol as membrane stabilizer (“vesicular cement”), enhancing the rigidity of the proniosomal bilayer and the entrapment efficiency of the vesicles; (c) lecithin acting as membrane stabilizer and also as skin permeation enhancer; (d) alcohol as organic solvent, with important role on vesicles size and formation, and permeation rate of the active ingredient; and (e) aqueous phase for hydration and gel formation [21]. Commonly used non-ionic surfactants are: sorbitan fatty acid esters (e.g., Span 20, Span 40, Span 60, and Span 85), polyoxyethylene fatty acid esters (e.g., Tween 80, Tween 20, Tween 40, and Tween 60), and alkyl ethers (e.g., Brij 30, Brij 58, Brij 72, and Brij 76). For this study, a sorbitan fatty acid ester, namely Span 60, was selected due to its ability to provide stable proniosomes with high entrapment efficiency, as previous studies reported [20,32,33]. The most frequently used lecithins to obtain proniosomal systems are soy and egg lecithins. However, soy lecithin is preferred more often, as several studies demonstrated improved entrapment efficiency, enhanced stability, and favorable rheological features, likely due to its higher content of unsaturated phospholipids [34,35]. Therefore, in the present study, a refined soy phospholipid with 70% phosphatidylcholine (Lipoid S 75) was selected. For the alcohol phase, ethanol was chosen instead of other alcohols, such as propanol, isopropanol, and butanol, due to its high water miscibility, which favors efficient lipid hydration and promotes the formation of larger and more stable vesicular systems [36]. For the aqueous phase of the studied proniosomal gels, distilled water was selected [20].

Consequently, the formulation was designed based on well-established principles reported in the literature for proniosomal systems [34,37,38,39,40]. The selected ratios of the excipients (Table 1) fall within ranges commonly reported to yield stable proniosomal gels with satisfactory vesicular characteristics (e.g., size, homogeneity, and entrapment efficiency).

The concentration of SIL in the final product (SIL-PG) was 0.3%, which we previously employed for the encapsulation of rutin [31], and it also falls within the concentration range at which other skin formulations were found therapeutically active. For instance, Samanta et al. demonstrated that the topical application of a 0.2% SIL-loaded hydrogel exerted potent wound-healing activity in mice [17]. Moreover, a clinical study assessing the efficacy and safety of an antioxidant serum containing 0.5% silymarin showed that this formulation improved acne vulgaris severity [41].

SIL-PG was obtained through the coacervation phase-separation method. The formulation and preparation of SIL-PG were followed by a thorough assessment of its organoleptic, physicochemical, and pharmaceutical characteristics, indicating the proper encapsulation of the bioactive agent into the gel matrix and that its pH, rheological behavior, spreadability, consistency, in vitro drug release, and permeation properties are suitable for dermal application.

#### 2.1.1. Organoleptic Properties, pH, and Entrapment Efficiency

**Figure 1:**
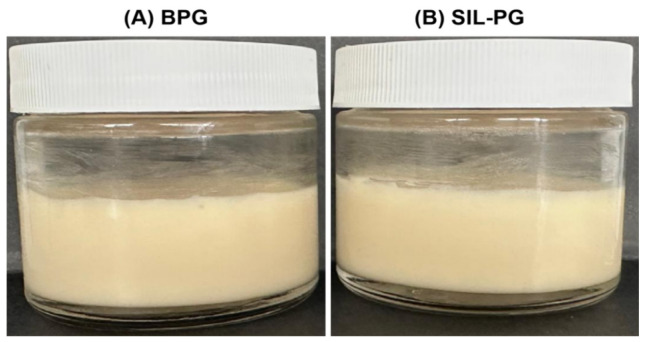
Physical appearance of the developed proniosomal gels: (**A**) blank proniosomal gel (BPG) and (**B**) silibinin-loaded proniosomal gel (SIL-PG).

Figure 1 comparatively illustrates the aspect of the two obtained gels, namely BPG and SIL-PG. With a strong macroscopic similarity between them, both BPG and SIL-PG presented a homogeneous and creamy appearance and a slight yellowish color.

The pH of the obtained formulations was further determined. The results indicated that the pH value of the BPG was 7.11 ± 0.09, as previously reported [31]. A slight increase to 7.57 ± 0.06 was observed when SIL was incorporated into the BPG matrix. These neutral values fall into the recommended pH range for semisolid preparations (between 4.5 and 8.5) by the European Pharmacopeia [31], while both BPG and SIL-PG are expected to be well-tolerated following skin application. The entrapment efficiency of SIL-PG was 57.00 ± 0.08%, which is comparable to values reported for related phytochemical-loaded proniosomal gels, such as puerarin-loaded proniosomal gel (62.00 ± 0.26%) and rutin-loaded proniosomal gel (>50%), supporting the adequate incorporation of SIL into the proniosomal matrix [21,31].

#### 2.1.2. Dynamic Light Scattering (DLS) Measurements

**Figure 2:**
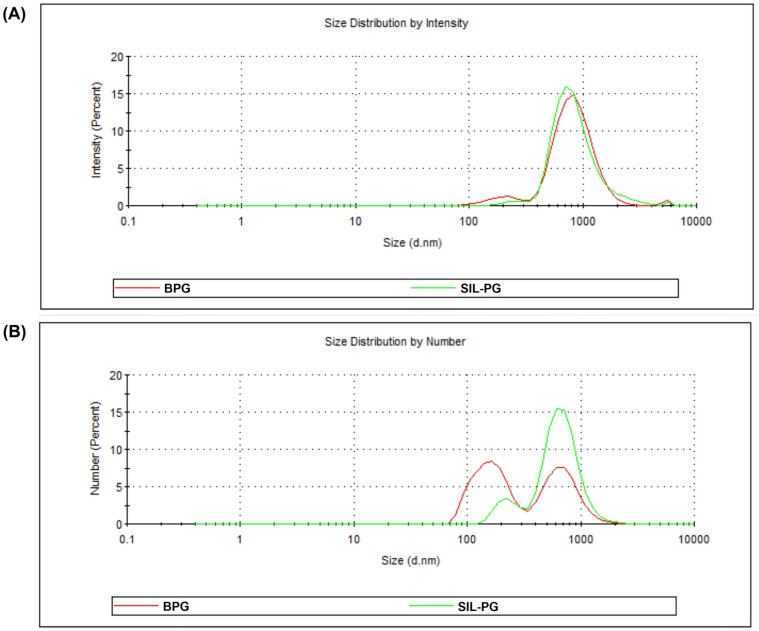
DLS analysis of BPG (red line) and SIL-PG (green line): (**A**) size distribution by intensity; (**B**) size distribution by number. The experiment was conducted using three independent replicates (*n* = 3).

DLS analysis (Figure 2) performed on the vesicles obtained from the hydrated formulations showed that BPG (red line) and SIL-PG (green line) exhibited PDI values of 0.43 ± 0.06 and 0.35 ± 0.01, respectively, denoting slightly polydisperse systems and broad vesicle size distribution following hydration [22]. Zeta (ζ) potential values were −58.0 ± 0.03 mV for BPG and −56.1 ± 1.01 mV for SIL-PG at neutral pH, being indicative of the electrostatic stability of the vesicular dispersions [42].

#### 2.1.3. FTIR Analysis

**Figure 3:**
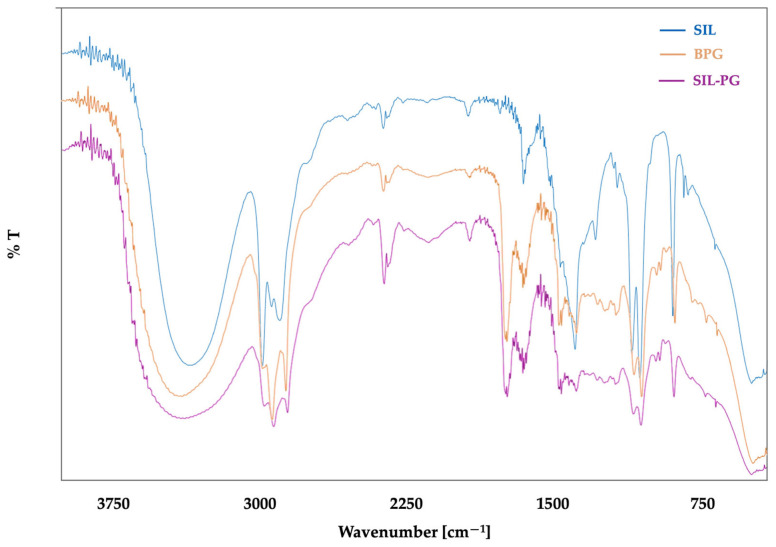
FTIR spectra of silibinin (SIL), blank proniosomal gel (BPG), and silibinin-loaded proniosomal gel (SIL-PG).

The FTIR spectra of SIL, BPG, and SIL-PG were compared as a complementary tool to investigate possible interactions between SIL and the proniosomal matrix and to assess the chemical compatibility of the components within the gel system (Figure 3). SIL exhibited its characteristic absorption bands, including a broad band in the 3600–3200 cm^−1^ region attributed to O-H stretching vibrations, absorption bands at 2972.30 cm^−1^ and 2887.43 cm^−1^ corresponding to C-H stretching, a signal at 1643.51 cm^−1^ associated with conjugated carbonyl and/or aromatic C=C vibrations, and intense bands at 1089.78 cm^−1^ and 1051.20 cm^−1^ related to C-O-C stretching vibrations.

BPG showed its own characteristic absorption profile, with major bands at 2920.20 cm^−1^, 2850.64 cm^−1^, 1732.02 cm^−1^, 1643.51 cm^−1^, 1467.69 cm^−1^, 1379.10 cm^−1^, and 1049.27 cm^−1^. It should be noted that several absorption bands observed in the SIL spectrum were also present in the blank formulation, particularly in the O-H, C-H, carbonyl/aromatic C=C, and C-O/C-O-C regions. This overlap may be attributed to the presence of similar functional groups in SIL and in the excipients composing the proniosomal gel matrix. Thus, these common bands cannot be considered as specific markers for SIL in the SIL-PG spectrum. In the spectrum of SIL-PG, the overall absorption profile was largely influenced by the blank gel matrix, and some SIL-related bands were overlapped or masked by BPG signals. Consequently, the FTIR data should be interpreted mainly as supportive evidence of chemical compatibility and possible intermolecular interactions, rather than as definitive evidence of SIL incorporation into the formulation. Slight shifts in band position, band broadening, and intensity changes were noted, especially in the O-H stretching and fingerprint regions, which may suggest possible intermolecular interactions between SIL and the gel matrix, most likely through hydrogen bonding; however, these changes should be interpreted with caution due to the extensive overlap between SIL and BPG bands. Importantly, the absence of major new absorption bands, together with the lack of disappearance of key characteristic bands, suggests that no evident chemical incompatibility or major chemical transformation occurred during SIL loading into the gel.

#### 2.1.4. Rheological Properties

**Figure 4:**
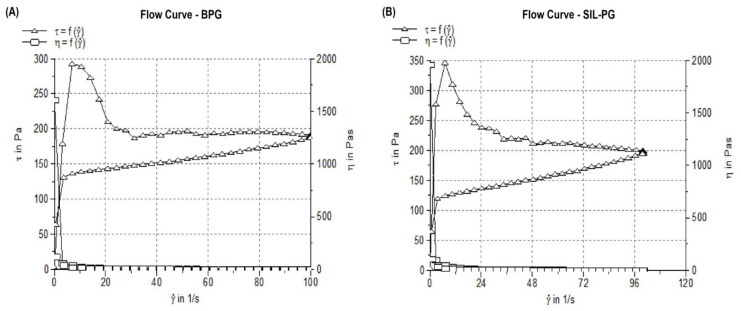
Flow and viscosity profiles of the experimental proniosomal gels: (**A**) blank proniosomal gel (BPG) and (**B**) silibinin-loaded proniosomal gel (SIL-PG). The experiment was conducted using three independent replicates (*n* = 3).

The next phase consisted of a series of analyses to evaluate the rheological behavior of the developed formulations, namely BPG and SIL-PG. As presented in Figure 4, the flow and viscosity profiles of the tested gels revealed non-Newtonian, pseudoplastic (shear-thinning) properties, as their viscosity decreased with increasing shear rate. Similar pseudoplastic behavior was also demonstrated by other studies on proniosomal gels with the same qualitative composition of the gel matrix [22,31,40].

The distinct “spur” present at the start of flow (292.7 Pa at 6.972 1/s for blank formulation and 345.8 Pa at 7.058 1/s for SIL-PG) on the upward segment of the rheograms (shear stress vs. shear rate) of the tested proniosomal gels (Figure 4) can indicate their structural anisotropy. Proniosomal gels are complex, highly concentrated vesicular systems, often consisting of non-ionic surfactants, cholesterol, and a small amount of solvent that form a dense, well-packed lamellar liquid crystal structure. When left undisturbed, proniosomal gels can form a highly organized, “stiff” network in which lamellar layers are aligned in a way that requires an extra force to overcome this state before steady-state flow could be achieved [43,44,45]. Also, the flow curves presented a hysteresis area, indicating the thixotropic behavior of the tested proniosomal gels. This complex flow behavior (pseudoplastic–thixotropic) is advantageous because it allows easy and even spreading of the proniosomal gel over the skin’s surface while ensuring that the product does not run off the skin after application. Moreover, the thixotropic properties facilitate preparation, use, and application to the skin due to the rapid formation of a homogeneous and stable film [21].

Fitting the flow and viscosity data to Ostwald de Waele and Herschel–Bulkley rheological models, it was observed that both mathematical equations best described the viscosity of tested gels, producing R^2^ values > 0.999 (Table 2). Instead, the low values of R^2^ obtained for the Ostwald de Waele and Herschel–Bulkley models (0.4998 and 0.5429) indicate that these time-independent and shear-rate-dependent, non-Newtonian models did not accurately describe the flow behavior of the studied proniosomal gels [46]. This can be attributed both to their heterogeneous structure, formed by an organized network of vesicles (primary vesicles and larger vesicular aggregates) dispersed within a gel matrix, and to their pronounced thixotropy [47]. The structure of proniosomal gels breaks down under shear but gradually recovers when shear decreases, due to time-dependent structural rearrangements that take place, producing non-linear regions in the flow curve and consequently deviations from model assumptions [31]. Moreover, for the SIL-PG formulation, SIL can further affect the microstructural organization, promoting non-linear flow behavior. The flow index values obtained with both models were much lower than 1, confirming the pseudoplastic character of the tested proniosomal gels. These results, based on regression analysis, are consistent with those reported by other previous studies [22,48]. Furthermore, no significant differences between the *n* values calculated for the BPG and SIL-PG formulations with each model can be observed. The consistency index values calculated for SIL-PG were slightly higher than those of BPG. Systems exhibiting pseudoplastic and non-Newtonian rheology indicate that the gel’s viscosity decreases under high shear conditions, a phenomenon known as shear-thinning, thus facilitating its topical application because it ensures uniform distribution on the skin [49,50].

The apparent viscosity of the SIL-PG formulation (1.97 ± 0.02 Pa∙s) was only 1.04-fold higher than that of the BPG formulation (1.89 ± 0.01 Pa∙s), whereas the thixotropy of SIL-PG (7477 Pa/s) was 1.58-fold higher than that of the BPG (4739 Pa/s). The variations in viscosity and especially the thixotropy degree can be explained by the influence of SIL on the BPG matrix. SIL, a polyphenolic flavolignan, presents a complex structure that allows it to interact with both polar and non-polar environments. Therefore, when encapsulated in a proniosomal gel system, SIL can interact with both vesicular bilayers and the surrounding gel matrix of the system. Through its phenolic and carbonyl groups, SIL can form hydrogen bonds with polar head groups of surfactants and cholesterol and water molecules, which reinforce the system’s structural network, resulting in increased viscosity and a more pronounced thixotropic character. As several studies already indicated, polyphenolic compounds, including SIL, are able to form hydrogen bonds with phospholipids (e.g., phosphatidylcholine), cholesterol, and other surfactants [51,52,53,54].

#### 2.1.5. Consistency Measurements

**Figure 5:**
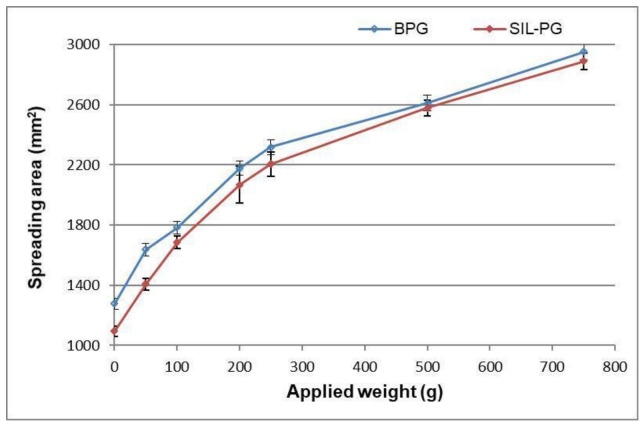
Spreading characteristics of experimental proniosomal gels: blank proniosomal gel (BPG) and silibinin-loaded proniosomal gel (SIL-PG). The experiment was conducted using three independent replicates (*n* = 3).

The following measurements focused on the consistency of the formulations developed in the study. The penetrometric test revealed the following penetration values: 233.0 ± 1.2 mm for BPG formulation (as was reported in our previously published study [31]) and 218.0 ± 16.6 mm for SIL-PG. As the penetration depth value produced by the SIL-PG was 1.07-fold lower than that of the BPG, one can suggest that it was slightly more consistent, respectively slightly harder, than the blank formulation. Figure 5 shows that the spreading areas for both formulations increase dependently on the applied weight. Moreover, the spreading areas produced by the SIL-PG formulation were slightly lower than those measured for the proniosomal gel matrix, especially in the range of 0–250 g of the applied weight; after that, the differences between the spreading values became insignificant, and the spreadability profiles of BPG and SIL-PG formulations were almost superimposable.

The calculated values of the spreading areas showed good spreadability of the experimental proniosomal gels and fell within the range reported by other authors for this category of modern semisolid vehicles [55,56]. Penetrometric and parallel-plate results, revealing the consistency of the tested proniosomal gels, indicated strong correlation with the outcomes of the steady-shear flow test.

#### 2.1.6. The Selection of the Receptor Medium for In Vitro SIL Release and Permeation Studies

According to the recommendations of regulatory agencies, the composition of the receptor medium to be used for in vitro drug release/permeation tests from topical dosage forms is selected based on “sink” conditions requirements (the solubility of the drug should be from 3 to 10 times higher than the maximum attainable concentration in the receptor compartment), so that the release/permeation of the drug is not hindered [57,58]. Since SIL is a poorly water-soluble drug, a simple buffer solution, PBS of pH 7.4, commonly used for in vitro drug release/permeation tests, does not ensure sink conditions; addition of surfactants or organic solvents is required to enhance its solubility. Therefore, nine receptor solutions consisting of PBS of pH 7.4 in combination with 0.5% (*w*/*w*) Eumulgin B 2 PH, Brij^®^ 35 or Brij^®^ S20, 1% (*w*/*w*) Tween 80 or Tween 20, 30% (*w*/*w*) ethanol, and 20, 30, or 60% (*w*/*w*) diethylene glycol monoethyl ether were tested for their ability to solubilize SIL. Also, the minimum solubility value of SIL in the receptor medium necessary to meet “sink” conditions was calculated based on the amount of proniosomal gel (approx. 300 mg) placed in the donor chamber of the vertical diffusion cell. The calculated amount of SIL was approx. 0.9 mg (for a theoretical content of 3 mg/g), which led to a concentration of 0.138 mg/mL SIL for instantaneous release in the 6.5 mL receptor medium. Hence, the minimum limit of SIL solubility in receptor medium should be between 0.414 and 1.380 mg/mL. Table 3 lists the solubility values of SIL in the nine receptor solutions.

The solubility values in receptor solutions containing surfactants and ethanol were very low, indicating their poor ability to solubilize SIL, and are not in compliance with the required “sink” conditions. Instead, the three receptor solutions containing diethylene glycol monoethyl ether of various concentrations showed a higher ability to solubilize SIL (Table 3). Moreover, the solubility values of SIL in these three receptor solutions are higher than the minimum solubility limit mentioned above (0.414 mg/mL), so they can be used as receptor medium for the in vitro SIL release/permeation tests. Although the mixture of PBS of pH 7.4 with 60% diethylene glycol monoethyl ether produced the highest solubility value, it was not selected as a receptor medium considering that a 60% concentration of diethylene glycol monoethyl ether can compromise the integrity of the synthetic membrane or of the skin barrier [57,58,59,60]. Consequently, for the SIL in vitro release test, phosphate-buffered saline pH 7.4 with 30% diethylene glycol monoethyl ether was selected as a receptor medium, and phosphate-buffered saline pH 7.4 with 20% diethylene glycol monoethyl ether was used for the in vitro permeation test.

#### 2.1.7. In Vitro Drug Release and Skin Permeation Studies

(a) In vitro drug release studies

In vitro release test is an important tool for the development of semisolid dosage forms, as it reveals the product performance and the potential to deliver high levels of drug at the site of application. Also, this test can indicate the cumulative effects of various physicochemical and rheological properties of the formulation on the product performance [60,61].

**Figure 6:**
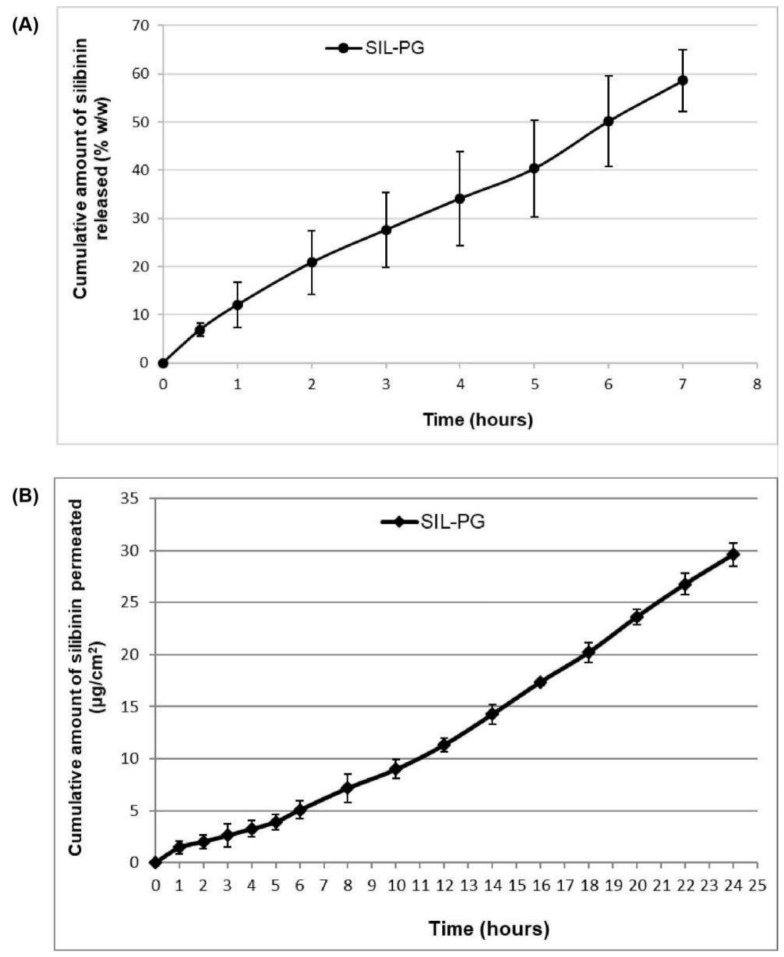
The cumulative kinetic profiles of SIL release (**A**) and permeation (**B**) from the investigated proniosomal gel (mean ± SD, *n* = 5): cumulative amount of SIL released in % (**A**) and in µg/cm^2^ (**B**) versus time.

Figure 6A shows the cumulative amounts of SIL released from the experimental proniosomal gel through the hydrophilic synthetic polyethersulfone membrane in the receptor medium (solution of PBS of pH 7.4 with 30% diethylene glycol monoethyl ether) after 7 h of testing. Table 4 shows the values of the SIL release parameters (steady-state flux, J_ss_; permeability coefficient, K_p_; and release rate, k) calculated based on the obtained experimental data.

Figure 6A shows that the cumulative percentages of released SIL through the synthetic membrane increased from 6.89 ± 1.32 (after 30 min) to 58.61 ± 6.46 (after 7 h). The SIL transfer through the synthetic membrane was relatively slow, as indicated by the steady-state flux and release rate values (Table 4). The profile of the cumulative release plot indicates that during the testing period, established based on the official recommendations, the steady state was not achieved. This suggests that the formulation exhibited a moderate extent of SIL release under the investigated experimental conditions. The lack of steady state on the cumulative release profile was observed in another study performed on creams containing SIL as the main constituent of silymarin [62].

To evaluate the in vitro release profiles of SIL from proniosomal gel, the experimental data were analyzed using four kinetic models, and the fitting results are listed in Table 5.

As can be observed, the release profile of SIL from the proniosomal gel through the polyethersulfone membrane fitted best to the Korsmeyer–Peppas model, for which the highest determination coefficient was calculated (R^2^ = 0.9993). In this model, the value of the diffusion exponent, n, reveals the possible drug release pattern: if n ≤ 0.45, Fickian diffusion governs the drug release; if 0.45 < n < 0.89, drug release follows an anomalous non-Fickian transport; if n ≥ 0.89, case II transport controls the drug release. In the case of the SIL-PG formulation, the calculated diffusion exponent value (0.77) suggests an anomalous non-Fickian transport (a combination of diffusional and relaxational transport).

(b) In vitro skin permeation studies

The specific permeation profile of SIL from the experimental proniosomal gel through porcine ear skin is illustrated in Figure 6B. The slow and sustained permeation of SIL is suggested by the low values of transcutaneous flux (1.24 ± 0.04 μg/cm^2^/h) and permeation rate (7.33 ± 0.26 μg/cm^2^/h^1/2^) (Table 4); after 24 h of testing, only 29.61 ± 1.08 μg/cm^2^ of active ingredient permeated. In addition, the transcutaneous permeation profile of SIL from the proniosomal gel showed a lag time of 1.46 ± 0.61 h (Table 4), as can be expected for slow diffusion. These results are consistent with those previously reported by other authors [62]. Sustained skin permeation of an active ingredient is considered beneficial for topical semisolid formulations because it can ensure prolonged drug release and extended residence time at the site of application, improving therapeutic efficacy and reducing the frequency of administration [63].

Fitting the in vitro permeation data of the SIL-PG formulation to different kinetic models and analyzing the results (Table 5), the highest value of the determination coefficient was obtained for the zero-order model (R^2^ = 0.9836), suggesting a constant permeation rate of SIL from the proniosomal gel, independent of the amount of active ingredient remaining in the delivery system.

The experimental results of the in vitro release and permeation tests can be attributed to the concomitant effects of several factors, such as: the SIL solubility in the formulation components; the SIL distribution among the oily and aqueous regions of the proniosomal vesicles; and the characteristics of the proniosomal matrix (e.g., vesicle size, viscosity, and consistency).

Similar observations have been confirmed by other authors for different topical SIL-based formulations, reporting a favorable and controlled drug release following encapsulation. Specifically, Zadeh et al. reported a sustained release of SIL from loaded polymeric micelles with 40% of the compound being released within the first 10 h and 70% during the first 48 h, which translated into a proper depot effect [64]. Additionally, Tan et al. presented the controlled and pH-dependent in vitro release of SIL from a carbon nanotube-based delivery system that might be beneficial for enhancing therapeutic efficiency while reducing side effects [54]. Regarding proniosomal platforms, there are previous reports explicitly outlining the sustained release profiles of entrapped drugs. Thus, the encapsulation of a palm tocotrienol-rich fraction into proniosomal gel resulted in a low and slow initial release of the drug due to the barrier-like behavior of the niosomal bilayers that conferred controlled release, which is preferred for reducing application frequency, improving patient compliance, and maintaining steady drug levels [22]. In another study, it was found that curcumin presents an immediate in vitro release from the reference dispersion, with around 90% of the drug being released within the first 2 h, while its formulation as a proniosomal gel leads to a significant sustained release pattern over a period of 24 h [23].

Hence, SIL loading into proniosomal gel may be advantageous due to its gradual release from the formulation. However, pure SIL suspension and non-gellified niosomal controls were not experimentally included in our study, which is acknowledged as a limitation that will be addressed in future studies to clearly distinguish the contribution of the free compound, gel matrix, and vesicular incorporation to the release and permeation profiles of SIL-PG.

### 2.2. Skin Biocompatibility Evaluation Using 2D and 3D In Vitro Models

Two-dimensional (2D) in vitro models consisting of monolayer cultures still represent a widely employed method for safety evaluations and play a major role in predicting dermal toxicity [65]. Considering that keratinocytes constitute the predominant cellular component of the epidermis and are highly prone to the application of topical treatments due to their outermost localization [66,67], we initially aimed at an in vitro evaluation of SIL-PG using the HaCaT cell line, which represents a validated experimental model of human immortalized keratinocytes extensively employed not only for the study of epidermal homeostasis and pathophysiology but also for the evaluation of potential skin irritation, sensitization, and toxicity caused by chemicals and topical formulations [68,69,70,71].

The concentration range (300, 400, and 500 μg/mL) selected for biological testing corresponds to the ones previously reported for the screening of topical gel-based formulations loaded with botanical compounds [72,73]. To clearly portray the influence of the gel matrix on the cytocompatibility of the bioactive compound, the assays included BPG (at the same concentrations used for SIL-PG—300, 400, and 500 μg/mL) and free SIL (at concentrations equivalent to those in SIL-PG—0.9, 1.2, and 1.5 μg/mL).

#### 2.2.1. Cell Viability Assessment

**Figure 7:**
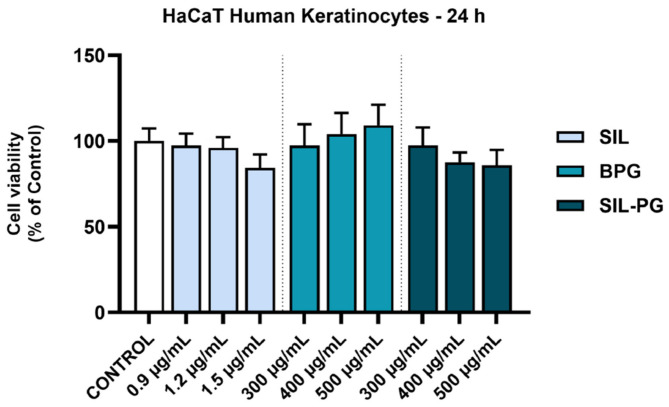
Graphical representation of the cell viability percentages (%) in HaCaT cells after 24 h treatment with silibinin (SIL) at concentrations of 0.9, 1.2, and 1.5 µg/mL, blank proniosomal gel (BPG), and silibinin-loaded proniosomal gel (SIL-PG) at concentrations of 300, 400, and 500 µg/mL. Results are presented as percentages normalized to control (HaCaT cells without treatment) and are expressed as mean values ± standard deviation of three different experiments performed in triplicate (*n* = 3). One-way ANOVA and Dunnett’s post-test were applied to assess the statistical significance of the results.

The viability of HaCaT cells following the 24 h treatment with SIL (0.9, 1.2, and 1.5 μg/mL), BPG, and SIL-PG (300, 400, and 500 μg/mL) is presented in Figure 7. Regarding SIL, the results indicated a concentration-dependent decrease in viability to 97.3% (at 0.9 μg/mL), 96.1% (at 1.2 μg/mL), and 84.3% (at 1.5 μg/mL). This result ties well with previous reports evaluating the cytotoxicity of SIL or SIL-based formulations in healthy cells. For instance, Dehelean et al. found that the cytotoxic properties of SIL follow a dose- and cell type-dependent trend after 24 h of treatment, with a stimulatory activity at low concentrations and an inhibitory effect at high concentrations. In HaCaT cells, they reported an increase in cell viability at 1 μM and a loss of viability starting with the concentration of 25 μM [74]. In another paper aimed to evaluate the cytotoxicity of SIL (25–200 µM) in healthy HGF-1 cells, the MTT test showed that this concentration range does not affect cell viability after 48 h of treatment, with the highest concentration of 200 µM reducing the percentage of viable cells to only 83.48% [75].

On the contrary, BPG treatment caused a gradual stimulation of cell viability that increased from 97.3% (at 300 μg/mL) to 104.0% (at 400 μg/mL) and 109.1% (at 500 μg/mL). Comparatively, a study conducted by Chavan et al. found minimal cytotoxicity of the plain proniosomal gel (0.1, 1, 10, and 100 μg/mL) in L929 and HaCaT cells after 24 h of treatment [76]. Parallel evidence also supports the biocompatibility of blank semisolid carriers (i.e., oleogels and hydrogels) in skin-derived cells at concentrations comparable to the ones tested herein [72,73,77].

SIL-PG yielded similar results to SIL, with cell viability percentages slightly declining to 97.5%, 87.6%, and 85.8% at 300 μg/mL, 400 μg/mL, and 500 μg/mL, respectively. The high similarity between the response of HaCaT cells to SIL and SIL-PG could indicate that the incorporation of this active ingredient into BPG does not cause additional cytotoxicity, preserves the biological properties of SIL, and ensures proper delivery of the compound to the cells. A recent study demonstrated that the incorporation of SIL into magnetic niosomal nanoparticles improves its cytotoxicity in HT-29 colon cancer cells, while presenting no significant effect on the effect on HEK-293 cells’ viability after 24 and 48 h of treatment [78]. Parvanescu et al. showed that the loading of plant-derived compounds in oleogels leads to compound-dependent effects on the HaCaT cell viability. Namely, after 24 h, the betulin-loaded oleogel reduced viability at low concentrations and increased it over 100% at the highest tested concentration (500 μg/mL), while the lupeol-loaded oleogel triggered a gradual reduction in cell viability [73].

The changes in HaCaT cell viability percentages following SIL, BPG, and SIL-PG treatments were not significant compared to control and exceeded 70%, which, according to ISO 10993-5:2009 standards classifying samples as cytotoxic when viability drops by more than 30% [79], suggests they lack toxic effects in HaCaT cells at the tested concentrations and treatment time.

#### 2.2.2. Cell Morphology and Confluence Evaluation

**Figure 8:**
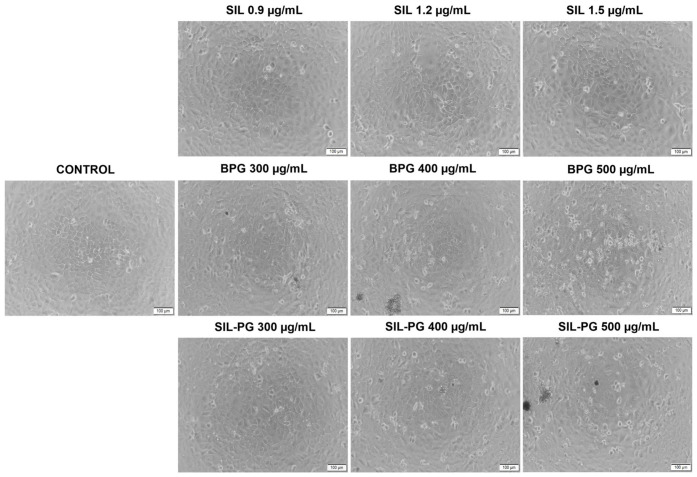
Representative images showing the morphology and confluence of HaCaT cells after 24 h treatment with silibinin (SIL) at concentrations of 0.9, 1.2, and 1.5 µg/mL, blank proniosomal gel (BPG), and silibinin-loaded proniosomal gel (SIL-PG) at concentrations of 300, 400, and 500 µg/mL. The images were captured in brightfield at a 20× magnification, and the scale bars indicate 100 µm. The experiment was performed three times in triplicate (*n* = 3).

Given that cell morphology analysis provides valuable information about cell health and status, allowing early identification of cell responses to external factors [21,80], the next step in evaluating the in vitro safety profile of SIL, BPG, and SIL-PG was the microscopic evaluation of the HaCaT cells’ shape, size, and confluence after 24 h of treatment. As depicted in Figure 8, the applied treatments caused no significant stress to this cell line, with morphology and confluence being very similar to control. The highest concentration of BPG (500 μg/mL) led to a slight increase in round, floating cells, an aspect that can be related to the observed stimulatory effect of this treatment on cell viability, leading to overcrowding and subsequent detachment of some cells from the plate. No characteristic signs of cytotoxicity (e.g., shrinkage, reduced confluence, blebbing, changes in size, or morphology) were noticed.

Comparatively, at a higher concentration (100 µM), SIL caused a slight reduction in the confluence of HepaRG cells while preserving the morphology of H9c2(2-1) and HaCaT cells [74]. We have previously found that neither blank oleogel nor betulinic acid-loaded oleogel (100, 300, 500 µg/mL) induced any cell dismorphology or confluence loss in HaCaT keratinocytes and JB6 Cl 41-5a epidermal cells after 24 h of treatment [72].

#### 2.2.3. Colony-Formation Assay

The study continued by evaluating the impact of SIL, BPG, and SIL-PG on the clonogenic activity of HaCaT cells. The colony-formation assay reflects the ability of a cell within a population to grow into a colony, essentially assessing in vitro cell survival and division. It is a method employed to identify the cytotoxic potency of tested agents and can be a measure of in vitro tissue regeneration capability [81,82,83]. Specifically, this assay was previously used to assess the proliferative capacity of epidermal keratinocytes and dermal fibroblasts [84].

**Figure 9:**
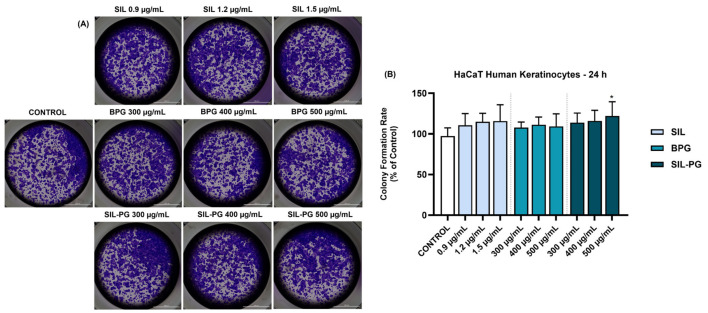
(**A**) Representative images depicting crystal-violet-stained HaCaT colonies after treatment with the samples of interest, silibinin (SIL), blank proniosomal gel (BPG), and silibinin-loaded proniosomal gel (SIL-PG). The scale bars indicate 2000 µm. (**B**) Graphical representation of colony-formation rate (%) after treatment with blank proniosomal gel (BPG), silibinin (SIL), and silibinin-loaded proniosomal gel (SIL-PG). BPG and SIL-PG were tested at concentrations of 300, 400, and 500 µg/mL, while SIL was tested at concentrations of 0.9, 1.2, and 1.5 µg/mL. Results are presented as percentages normalized to control (HaCaT cells without treatment) and are expressed as mean values ± standard deviation of three different experiments performed in triplicate (*n* = 3). One-way ANOVA and Dunnett’s post-test were applied to assess the statistical significance of the results (* *p* < 0.05).

As illustrated in Figure 9, it can be noticed that SIL, BPG, and SIL-PG did not reduce colony formation or impact their development. On the contrary, a stimulatory effect at all tested concentrations was obtained, with a significant increase in colony-formation rate compared to control being registered only for SIL-PG 500 μg/mL. Thus, SIL increased the clonogenic rate in a concentration-dependent manner from 108.5% (at 0.9 μg/mL) to 112.1% (at 1.2 μg/mL) and 115.4% (at 1.5 μg/mL), BPG to 109.6% (at 300 μg/mL), 114.3% (at 400 μg/mL), and 110.5% (at 500 μg/mL), and SIL-PG to 113.9% (at 300 μg/mL), 115.9% (at 400 μg/mL), and 121.9% (at 500 μg/mL). These findings revealed that the samples have no long-term impairment on keratinocyte function, promote their clonogenicity in vitro, and potentially support epidermal regeneration and renewal. As the strongest effect was retained by SIL-PG, it can be suggested that the incorporation of SIL in BPG preserves its cytocompatibility in HaCaT cells, while slightly enhancing its pro-clonogenic properties in this cell line.

Although prior research on the impact of SIL on the colony-formation ability of healthy cells is scarce, there are multiple studies demonstrating its inhibitory effect on the clonogenic potential of tumor cells at concentrations higher than those tested herein [73,85].

#### 2.2.4. Nuclear and Mitochondrial Staining

As complementary information on the potential cytotoxicity of SIL, BPG, and SIL-PG in HaCaT cells, this work included an analysis of the aspect of nuclei and mitochondria in keratinocytes exposed to these samples for 24 h. Cell death is associated with specific morphological changes at the nuclear level that are indicative of its initiation and allow the differentiation between apoptosis, a programmed cell death that leads to chromatin cleavage, constriction, and fragmentation into pyknotic bodies, and necrosis, highlighted by nuclear swelling and inflammatory response [86]. On the other hand, mitochondria are highly important organelles, serving multiple cell functions, including energy generation and cell death modulation via various pathways such as apoptosis, pyroptosis, and ferroptosis [87].

**Figure 10:**
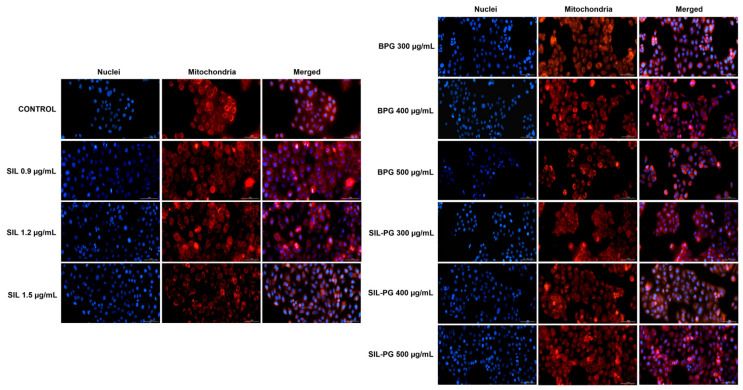
Representative images showing the nuclei and mitochondria of HaCaT cells after 24 h treatment with silibinin (SIL) at concentrations of 0.9, 1.2, and 1.5 µg/mL, blank proniosomal gel (BPG), and silibinin-loaded proniosomal gel (SIL-PG) at concentrations of 300, 400, and 500 µg/mL. The images were captured in brightfield at a 20× magnification, and the scale bars indicate 100 µm. The experiment was performed three times in triplicate (*n* = 3).

As illustrated by the images in Figure 10, the aspect of nuclei was constant across treatments and similar to control. They presented a round or oval shape, even blue signal, and a similar size in both the control and treated HaCaT cells, suggesting a comparable degree of viability between groups and a lack of cytotoxicity or cell death at the tested concentrations of SIL, BPG, and SIL-PG. Similarly, the tested samples did not present any visible signs of mitochondrial dysfunction, their morphological features being comparable to those of the control. They presented a network-like and even distribution within the intracellular compartment of both untreated and SIL, BPG, and SIL-PG-treated keratinocytes, which suggests a preserved mitochondrial function and metabolically active cells.

Overall, these results confirm that the loading of SIL in BPG maintains its safety profile in HaCaT cells, without mitochondrial-mediated cytotoxicity and cell death. We have noticed a similar trend in the case of betulinicacid-loaded oleogel (500 μg/mL) that caused no nuclear or mitochondrial impairments in HaCaT and JB6 Cl-5a skin cells after 24 h of exposure [72]. In fact, a previous study by Tie et al. reported that SIL exerts innate cytoprotective effects by preventing H_2_O_2_-induced oxidative stress and mitochondrial dysfunction in SH-SY5Y cells [88], while Wang et al. found that SIL safeguarded HaCaT keratinocytes against UVB-triggered apoptosis and mitochondrial injury [89].

#### 2.2.5. Spheroid Viability, Morphology, and Size

Despite their innate value in early dermal toxicity screening [68], conventional HaCaT 2D cultures are unable to completely replicate cutaneous complexity and cellular heterogeneity [90]. There is a notable development of 3D in vitro skin models for risk assessment that confer a more realistic overview of human skin structure and function and can more efficiently predict in vivo responses compared to traditional methods [65,91]. Thus, the next section of this research focused on validating the biocompatibility of the developed SIL-PG using a 3D HaCaT spheroid-based model. Spheroids are cell aggregates that organize into a 3D spherical structure and are extensively used to assess drug efficiency, permeation, or toxicity [91]. As an alternative in vitro skin model, spheroids can be formed of either fibroblasts or keratinocytes [92].

**Figure 11:**
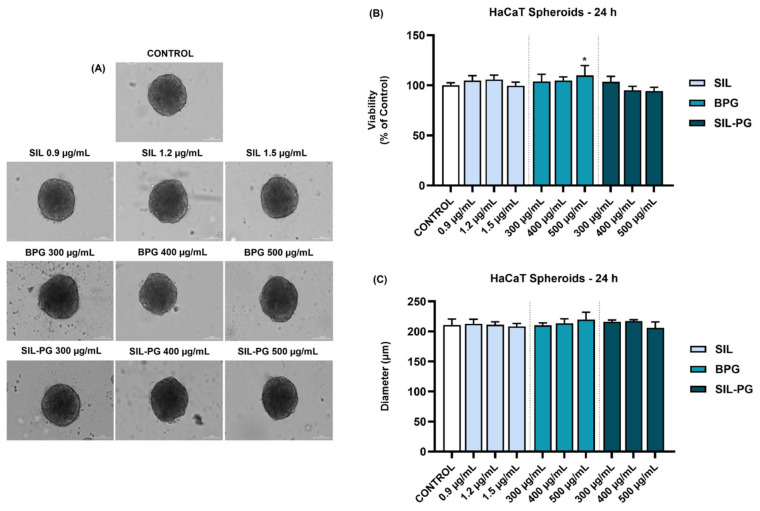
(**A**) Morphological features, (**B**) viability (% of control), and (**C**) diameter of HaCaT spheroids (μm) following a 24 h exposure to silibinin (SIL) at concentrations of 0.9, 1.2, and 1.5 μg/mL, blank proniosomal gel (BPG), and silibinin-loaded proniosomal gel (SIL-PG) at concentrations of 300, 400, and 500 µg/mL. The images were captured in brightfield at a 4× magnification, and the scale bars indicate 100 µm. Results are presented as percentages normalized to control (HaCaT spheroids without treatment) and are expressed as mean values ± standard deviation of three different experiments performed in triplicate (*n* = 3). One-way ANOVA and Dunnett’s post-test were applied to assess the statistical significance of the results (* *p* < 0.05).

Figure 11 summarizes the impact of a 24 h treatment with SIL, BPG, and SIL-PG on the structure, viability, and size of the formed HaCaT spheroids. Control spheroids presented a clear and well-defined round shape, a compact structure, and a diameter of around 200 μm. The applied treatments caused no visible impairments on spheroid morphology, as their size, shape, and integrity were maintained following all applied treatments. The viability of HaCaT spheroids changed in a treatment- and concentration-dependent manner. Namely, SIL increased viability to 104.7% and 105.8% at 0.9 μg/mL and 1.2 μg/mL, respectively, while retaining it at a value similar to control (at 99.7%) at 1.5 μg/mL. BPG caused a gradual increase in viability from 103.8% at 300 μg/mL to 104.7% at 400 μg/mL and 110.0% at 500 μg/mL, the latter value reaching statistical significance compared to control. At 300 μg/mL, SIL-PG enhanced viability to 103.6%, while at 400 μg/mL and 500 μg/mL, it slightly declined to 95.1% and 94.3%, respectively.

Interestingly, the viability of HaCaT spheroids following SIL, BPG, and SIL-PG treatments showed the same trend as that of HaCaT cells (Figure 7), indicating, on one hand, that the developed 3D system is treatment-responsive, and on the other hand, that the biocompatibility of the tested samples is maintained across both simple and complex in vitro models. Nonetheless, the spheroids showed slightly lower sensitivity compared to the 2D cell model, which is explained by their 3D architecture.

#### 2.2.6. Skin Irritation Test (SIT)

Even though HaCaT cells present wide applications and similar differentiation properties as normal keratinocytes [93], they were found unable to form a barrier, which is characteristic of the epidermis [94]. Therefore, the last part of the in vitro safety screening of SIL-PG focused on its potential irritant effect in EpiDerm™ reconstructed human tissues, consisting of human-derived keratinocytes organized in a 3D structure and serving as an advanced alternative to animal safety testing. The SIT performed on these inserts was designed to accurately predict the cutaneous irritant potential of chemicals, including cosmetic and pharmaceutical ingredients [95,96,97].

**Figure 12:**
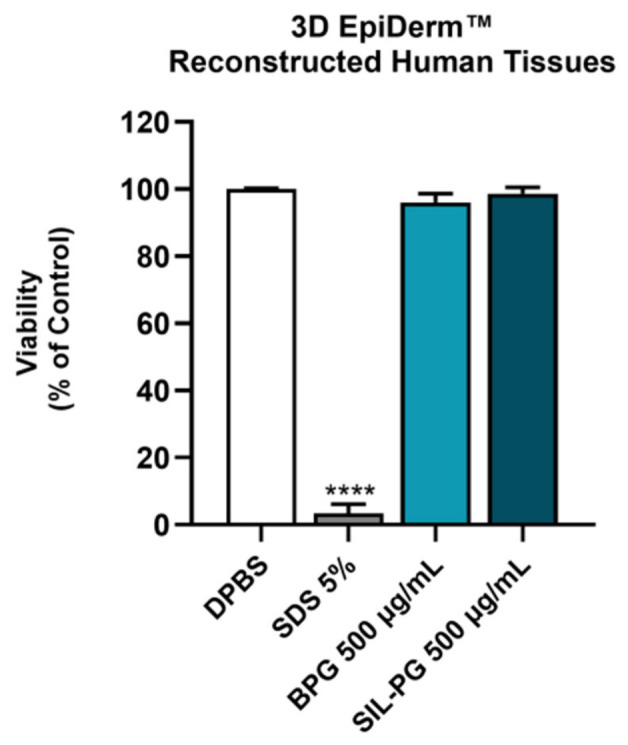
Graphical representation of the viability percentages of EpiDerm reconstructed human tissues (EPI-200 SIT) 18 h following treatment with blank proniosomal gel (BPG) and silibinin-loaded proniosomal gel (SIL-PG) 500 µg/mL. The positive control is represented by SDS 5%, and DPBS represents the negative control. A one-way ANOVA test was conducted to determine the statistical differences between the control group and the treated groups, followed by Dunnett’s multiple comparison post hoc test. (**** *p* < 0.0001). The experiment was conducted using three independent replicates (*n* = 3).

Figure 12 illustrates the results obtained from the exposure of 3D EpiDerm™ Reconstructed Human Tissues to DPBS (negative control), 5% SDS (positive control), BPG, and SIL-PG 500 μg/mL. As expected, 5% SDS caused a massive decrease in viability to 3.4%. The application of BPG and SIL-PG to the inserts did not affect their viability, with percentages of 96.0% and 98.6%, respectively. This result classifies both formulations as non-irritant because the viability was maintained above 50%, which confirms the absence of irritation, according to the manufacturer’s recommendations [31].

Hung et al. found that the topical application of silymarin-derived components (i.e., silibinin, silydianin, and silychristin) for up to 24 h caused no skin irritation [98]. Regarding the irritant potential of SIL-based formulations, Samanta et al. [17] formulated a hydrogel loaded with 0.2% SIL for topical use. Their in vivo results showed that its application over a period of 8 days led to a 56.3% contraction of the wound in treated mice, concluding that the formulation exerts strong wound-healing activity with no signs of irritation or inflammation [14]. Similarly, Pinzaru et al. examined the toxicological profile of blank and rutin-loaded proniosomal gels using the 3D EpiDerm™ reconstructed human tissues, concluding that these products present a proliferative effect by stimulating tissue viability, are non-irritant to the skin, and lack phototoxic potential [31].

### 2.3. In Ovo Irritant Potential and Vascular Toxicity

**Figure 13:**
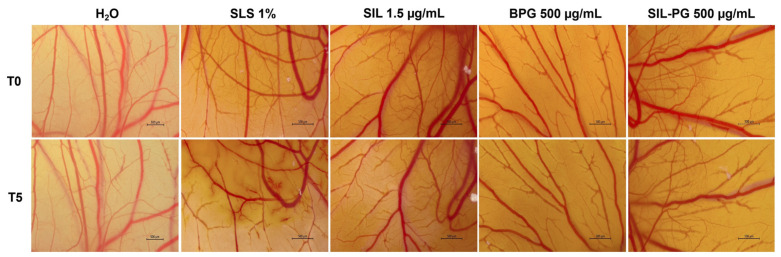
The aspect of the chorioallantoic membrane vasculature before treatment (T0) and 5 min after treatment (T5) with H_2_O (negative control), SLS 1% (positive control), SIL 1.5 μg/mL, BPG 500 μg/mL, and SIL-PG 500 μg/mL. The scale bars indicate 500 µm. The experiment was conducted using three independent replicates (*n* = 3).

The final step in the evaluation of the safety profile of the developed formulations was the assessment of their potential vascular toxicity and irritant effect in ovo, using the chorioallantoic membrane (CAM) as an experimental model. In this assay, SIL 1.5 μg/mL, BPG, and SIL-PG 500 μg/mL were directly applied to the CAM, which was monitored for 5 min for possible vascular changes. SLS at a concentration of 1% was used as a positive control, and H_2_O as a negative control. According to Figure 13 and Table 6, the results indicate that SLS 1%, as expected, acted as a severely irritating agent (IS = 19.94 ± 0.46), while H_2_O was non-irritant (IS = 0.07 ± 0.02). SIL of 1.5 μg/mL, BPG of 500 μg/mL, and SIL-PG of 500 μg/mL were classified as non-irritants as well, producing irritation score (IS) values of 0.07 ± 0.05, 0.45 ± 0.06, and 0.19 ± 0.05, respectively. These results suggest a lack of vascular impairments (hemorrhage, coagulation, and lysis) caused by the tested samples and good biocompatibility with the CAM.

In the same train of thought, other studies revealed that a proniosomal gel containing isoflavone, puerarin, and betulinic-acid-oleogel was non-irritant on the CAM [22,53]. Similarly, other herbal components intended for topical application (chlorogenic acid, apigenin, naringenin, and kaempferol) were assessed in terms of potential irritant capacity on the CAM [99].

## 3. Conclusions

The findings presented herein suggest that SIL-PG presents suitable organoleptic, physicochemical, and pharmaceutical properties for cutaneous application and an enhanced biosafety profile in vitro and in ovo, supported by a lack of cytotoxicity in HaCaT keratinocytes and spheroids and absence of irritant effect in 3D EpiDerm™ tissues and on the CAM. However, a limitation of this study is the clinical translation of the results. In fact, despite promising preclinical studies, clinical trials conducted on proniosomal formulations are generally limited, with extensive research being needed to complete the pharmaco-toxicological profile of such products [100]. Additionally, there are other aspects that should be thoroughly considered before their use in clinical practice, such as large-scale production and batch-to-batch reproducibility, long-term stability, cost-effective manufacturing, and regulatory approval [101]. Although the safety evaluation was performed using validated in vitro and in ovo models predicting the lack of skin toxicity and irritant effect of SIL-PG, they might not replicate entirely the in vivo behavior of the obtained formulation due to the complexity of the cutaneous barrier and potential local metabolic activities [102] that might interfere with the topical delivery of SIL, and thus urge the confirmation of the results obtained herein in extended studies. Moreover, the precise dermatologic and cosmetic indications of this formulation should be explored as future research directions that compile evidence of its applicability. Lastly, formulation and SIL loading optimizations should be considered to maximize the product’s pharmaceutical features and therapeutic efficacy, while the shelf life and stability (e.g., short-term, long-term, under accelerated conditions) of SIL-PG should be assessed before use.

## 4. Materials and Methods

### 4.1. Reagents

Silibinin, cholesterol, sorbitan monostearate (Span 60), and sodium lauryl sulfate (SLS) were bought from Sigma-Aldrich (Taufkirchen, Germany). Soya lecithin (Lipoid S 75) was kindly donated by Lipoid GmbH (Ludwigshafen, Germany), and ceteareth-20 (Eumulgin B 2 PH) was received as a free sample from BASF SE (Ludwigshafen, Germany). Polyoxyethylene lauryl ether (Brij^®^ 35), polyethylene glycol octadecyl ether (Brij^®^ S20), polysorbate 80 (Tween 80), and polysorbate 20 (Tween 20) were purchased from Merck KgaA (Darmstadt, Germany). Diethylene glycol monoethyl ether, 98%, was purchased from Thermo Scientific Chemicals (Geel, Belgium). Absolute ethanol, sodium chloride, potassium chloride, disodium phosphate, and monopotassium phosphate were provided by Chemical Company S.A. (Bucharest, Romania). Polyethersulfone hydrophilic synthetic membrane (Supor^®^ membrane, 0.45 µm and 25 mm) was supplied by Pall Corporation (Port Washington, NY, USA), and the pig skin was excised from pork ears provided by a local slaughterhouse. Distilled water was used to prepare the hydrogels, and bidistilled water was used to prepare phosphate-buffered saline pH 7.4. Cell culture reagents, namely Dulbecco’s Modified Eagle Medium (DMEM), phosphate-buffered saline (PBS), trypsin-EDTA solution, antibiotics (penicillin/streptomycin), and dimethyl sulfoxide (DMSO), were purchased from ATCC (Manassas, VA, USA). The MTT (3-(4,5-dimethylthiazol-2-yl)-2,5-diphenyltetrazolium bromide) kit was supplied by Roche Holding AG (Basel, Switzerland), while the staining reagents, Hoechst 33,342 and MitoTracker Red CMXRos, were delivered by Thermo Fisher Scientific (Waltham, MA, USA). Paraformaldehyde (4% solution in PBS) was procured from Santa Cruz Biotechnology (Dallas, TX, USA), and crystal violet (1% aqueous solution) was bought from Electron Microscopy Sciences (Hatfield, PA, USA).

### 4.2. Preparation of Proniosomal Gel Formulations

The proniosomal gels (BPG and SIL-PG) were prepared by the widely applied coacervation phase-separation technique [36,103]. In detail, accurately weighed amounts of active ingredient silibinin (0.3% *w*/*w*) and excipients (lipophilic non-ionic surfactant sorbitan monostearate—180 mg, cholesterol—30 mg, and soya lecithin—90 mg) were placed into a dry, wide-mouthed glass container. A total of 0.3 mL of absolute ethanol was added, the container was sealed with a stopper to prevent solvent loss, and the mixture was warmed to 60–70 °C in a water bath for approximately 5 min with shaking. This ensured all solid components, particularly the surfactant and cholesterol, dissolved completely. After that, 0.1 mL of pre-heated distilled water (at 60 °C) was added to the mixture, and warming was performed in the water bath at the same temperature, which continued for about 2–3 min with gentle stirring until a clear liquid was obtained. This mixture was then left to cool at room temperature, at which point it transformed into a white-yellowish, creamy proniosomal gel. The blank proniosomal gel was prepared following the same steps, but without incorporating SIL. Considering that the stability of proniosomal formulations might be altered by external factors (e.g., temperature and humidity), BPG and SIL-PG were stored in the dark, in well-closed containers at 4 °C during the experiments. No visual signs of instability (e.g., changes in color, consistency, or homogeneity [104]) were observed during storage.

### 4.3. Characterization of the Proniosomal Gels

#### 4.3.1. Organoleptic Properties and pH Measurement

The color, aspect, and homogeneity of BPG and SIL-PG were visually evaluated. To measure the pH of the proniosomal gels, a SevenExcellence™ S400-KIT pH meter (Mettler Toledo, Columbus, OH, USA) was used. Following a standard potentiometric protocol, the samples were prepared by dispersing 1 g of proniosomal gel in 20 mL of heated distilled water (at 50 °C and under stirring); the obtained aqueous dispersions were cooled and filtered. The pH was then measured directly from the filtrate, in triplicate, at 25 ± 2 °C.

#### 4.3.2. Entrapment Efficiency Calculation

For this assessment, SIL-PG (0.5 g) was initially dispersed in ultrapure water (5 mL). The obtained dispersion was filtered and centrifuged at 15,000 rpm for 15 min at 4 °C. The supernatant was further analyzed using the UviLine 9400 spectrophotometer (SI Analytics, Deutschland, Germany) by reading the absorbance at a wavelength of 288 nm. Entrapment efficiency (EE%) was calculated using the following formula:

(1) $$Entrapment\;Efficiency\;\left(EE\%\right)=\;\left(\;\frac{W_{SILinitial}-W_{SILfree}}{W_{SILinitial}}\;\right),$$

where W_SILinitial_ indicates the amount of the SIL in the formula, and W_SILfree_ indicates the amount of the SIL in the supernatant. This indirect approach is consistent with previously reported proniosomal gel protocols, in which the gel is dispersed or rehydrated in water or phosphate buffer, followed by centrifugation and spectrophotometric quantification of the non-entrapped fraction in the supernatant [21,39,105].

#### 4.3.3. DLS Analysis

DLS measurements were performed using a Zetasizer NanoZS (Malvern Panalytical Ltd., Malvern, UK) to determine the hydrodynamic diameter and zeta potential of BPG and SIL-PG. The DLS analysis was performed according to the method described by Liga et al. [21], with minor adaptations. Briefly, after dispersion of the gels, the samples were filtered through a 0.220 µm membrane filter and subsequently analyzed at a controlled temperature of 25 ± 2 °C using non-invasive backscatter (NIBS) detection at an angle of 173°. All measurements were carried out in triplicate for each sample (n = 3), and the results are expressed as mean ± SD.

#### 4.3.4. Fourier Transform Infrared (FTIR) Spectral Analysis

The functional groups of SIL, BPG, and SIL-PG were distinguished through FTIR analysis, with recordings performed in the 4000–400 cm^−1^ range. The instrument used for this analysis was the Bruker Vertex 70 spectrophotometer, Bruker Daltonik GmbH, equipped with a Platinum ATR spectrometer, Bruker Diamond Type A225/Q.I (Bremen, Germany).

#### 4.3.5. Rheological Measurements

The gels’ flow behavior and viscosity were evaluated by steady-state shear tests using a stress-controlled RheoStress 1 rheometer (Thermo Fisher Scientific, formerly Thermo Haake, Karlsruhe, Germany). The instrument was fitted with a titanium cone-plate system (C35/2Ti; 35 mm diameter, 2° angle, 0.105 mm gap) and a TCP/P Peltier unit to maintain a constant temperature of 23 °C. The analysis of experimental data was performed using HAAKE RheoWin Data Manager software (version 4.3). The measurements were performed in a controlled-rate mode, following a ramp-up and ramp-down protocol with a shear rate domain of 0.05 1/s to 100 1/s, 120 s for each ramp. The flow and viscosity profiles (shear rate, $\dot{\gamma }$ versus shear stress, τ, and viscosity versus shear stress) were fitted to the Ostwald de Waele and Herschel–Bulkley rheological models (Table 7), where τ is the shear stress (in Pa), K is the consistency index (in Pa∙s), $\dot{\gamma }$ is the shear rate, n is the flow behavior index, η is the viscosity (in Pa∙s), τ_0_ is the yield stress (in Pa), and η_0_ is the zero-share rate viscosity (in Pa∙s). If n < 1, the material presents a pseudoplastic (shear-thinning) behavior, and if n > 1, a shear-thickening behavior is attributed to the material. The lower the value of n, the more pseudoplastic the evaluated system is. The fit accuracy was analyzed based on the obtained value of the calculated correlation coefficient of the model. For evaluating the goodness of fit, the R-squared (coefficient of determination) parameter was considered.

To evaluate the thixotropy of the studied proniosomal gels, the method of the hysteresis area (the area between the upward and downward ramps) was used. The larger the hysteresis area, the more thixotropic the system is.

#### 4.3.6. Consistency Measurements

The characterization of the experimental proniosomal gels in terms of consistency was performed by two specific tests: the penetrometric test (method of European Pharmacopeia) [106] and the parallel-plate method [107]. The penetrometric test was performed to assess the hardness, which quantifies the structural strength retained by semisolid formulations. A penetrometer (PNR 12, Petrolab, Speyer, Germany), equipped with a microcone and a suitable container, was used for the assay. The test was conducted in accordance with the pharmacopeial protocol at a temperature of 25.0 ± 0.5 °C. The penetration depth was recorded in millimeters (mm) and presented as the arithmetic mean of 3 independent measurements ± SD. The hardness of the material is indirectly proportional to the cone’s penetration depth. The parallel-plate method was employed to evaluate the spreadability of the experimental proniosomal gels. A del Pozo Ojeda-SuñéArbussá extensometer was used, according to the literature [108]. Spreading profiles were constructed by plotting the surface areas (mean of 3 independent measurements ± SD) produced by the samples (at 23 ± 2 °C) versus applied weights.

#### 4.3.7. The Selection of the Receptor Medium for In Vitro SIL Release and Permeation Studies

To select the appropriate receptor medium for in vitro release and permeation studies, the solubility of SIL in nine mixtures of phosphate-buffered saline (PBS), pH 7.4, with different non-ionic surfactants or solvents was evaluated. The concentration of non-ionic surfactants was 0.5% (*w*/*w*) for Eumulgin B 2 PH, Brij^®^ 35 and Brij^®^ S20, and 1% (*w*/*w*) for Tween 80 and Tween 20. In the case of the used solvents, the concentration was 30% (*w*/*w*) for ethanol and 20, 30, and 60% (*w*/*w*) for diethylene glycol monoethyl ether. SIL solubility in the above-mentioned media was assessed using the saturation shake-flask method. An excess amount of SIL was added to a fixed volume of each medium (2 mL) in sealed vials to ensure saturation of the system. The vials containing the dispersions were continuously agitated at room temperature for 48 h to reach equilibrium. Then, the samples were centrifuged at 3000 rpm for 5 min to separate the undissolved solid. The supernatant (saturated solution) was separated from the sediment by filtration through a 0.45 µm membrane filter with a diameter of 25 mm (Teknokroma, Hennef, Germany). A sample of each filtered solution was diluted with absolute ethanol, and the SIL concentration was quantified spectrophotometrically (T70+ spectrophotometer, PG Instruments, Leicestershire, UK) in the UV domain at 294 nm. Three measurements were performed for each indicated solubility data.

#### 4.3.8. In Vitro Drug Release and Skin Permeation Studies

(a) In vitro drug release studies

A Franz diffusion cell assembly (Microette-Hanson system, model 57-6AS9, Chatsworth, CA, USA) was used to perform the drug release tests. The vertical diffusion cells had an effective diffusion area of 1.767 cm^2^ and a volume of the receptor compartment of 6.5 mL. A polyethersulfone hydrophilic synthetic membrane was used to separate the donor and acceptor compartments and was soaked in the receptor medium for 30 min prior to the test. Sink conditions throughout the in vitro release test were ensured by using PBS of pH 7.4 containing 30% diethylene glycol monoethyl ether as receptor medium. The acceptor compartment was filled with this medium (freshly prepared and deaerated) and separated from the donor compartment by the synthetic membrane. The donor chamber containing approximately 0.300 g proniosomal gel was fixed upon the membrane, avoiding air bubble incorporation. During the 7 h of the experiment, the Franz diffusion cell system was maintained at 32 ± 2 °C, and the receptor medium was constantly stirred at 600 rpm to avoid the effects of the diffusion layer. A total of 0.5 mL of the receptor fluid was withdrawn automatically at 0.5, 1, 2, 3, 4, 5, 6, and 7 h and replaced with an equal volume of fresh receptor medium to maintain sink conditions. The quantitative determination of SIL in the aliquots was carried out spectrophotometrically at a 329 nm wavelength, corresponding to the maximum absorption of the SIL in PBS of pH 7.4 containing 30% diethylene glycol monoethyl ether.

(b) In vitro skin permeation studies
  (b1) Preparation of skin membranes.

For the in vitro permeation studies, pig ear skin was used as a model membrane. The freshly excised pig ears were washed with tap water, and the hair was removed from the outer area of the ears. Using a dermatome (Acculan 3 TiElectroderma-tome, Aesculap B. Braun Company, Tuttlingen, Germany), the skin was excised at a thickness of about 500 μm. If the excised skin samples were not used immediately, they were kept at −18 °C for up to 2 months, and before use, the frozen samples were allowed to thaw at room temperature. Only the skin samples of satisfactory integrity and thickness (tested by visual examination and by measurement with a micrometer, respectively) were used in the permeation studies.

      (b2) Skin permeation experiments.

The in vitro permeation test was carried out as described above, but with some modifications: PBS of pH 7.4 containing 20% diethylene glycol monoethyl ether as receptor medium; the sampling times from the receptor chambers were at preset intervals of 1, 2, 3, 4, 5, 6, 8, 10, 12, 14, 16, 18, 20, 22 and 24 h. The SIL quantification in the samples was performed by UV spectrophotometric assay at the same wavelength (329 nm). The in vitro diffusion experiments were performed in five replicates.

(c) Data analysis of in vitro drug release and permeation studies

By applying linear regression to the average cumulative amount of SIL released/permeated per surface unit of membrane (μg/cm^2^) versus time (t, h), the steady-state flux (J_ss_, μg/cm^2^/h), the permeability coefficient (K_p_, cm/h), and the lag time (tL, h) were calculated. Similarly, the linear regression technique was used for the curve of the average cumulative amount of SIL released/permeated per surface unit of membrane (μg/cm^2^) versus the square root of time to calculate the release rate (k). In order to elucidate the predominant drug release mechanism, analysis of the obtained SIL in vitro release/permeation profiles was carried out by fitting the data to four kinetic models commonly used for topical systems.

- Zero order model:

M_t_ = M_0_ + K_0_t, (2)

where M_t_ represents the amount of drug dissolved during time t, M_0_ represents the initial amount of drug in solution (which is usually zero), and K_0_ represents the zero-order release constant expressed in units of concentration/time.

- First order model:

logC = logC_0_ − K_1_t/2.303, (3)

where C_0_ is the initial concentration of drug, K_1_ is the first-order release constant, and t is the time.

- Higuchi model:

M = K_H_t^1/2^, (4)

where M represents the amount of active substance released at time t, and K_H_ is the Higuchi release constant.

- Korsmeyer–Peppas model:

Mt/M_∞_ = K_P_t^n^, (5)

where Mt/M_∞_ represents the ratio of the amount of substance released at time t, K_P_ is the Korsmeyer–Peppas release rate constant, and n is the diffusion coefficient. In this case, the data corresponding to the release of 60% of the quantity of the substance were analyzed.

For each kinetic model, the goodness of fit was evaluated by linear regression analysis, calculating the R-squared (determination coefficient). The kinetic model providing the highest value of the determination coefficient was considered the most suitable for describing the SIL release from the studied proniosomal gel.

### 4.4. 2D and 3D In Vitro Studies

#### 4.4.1. Sample Preparation for In Vitro Testing

To conduct the in vitro assays, SIL, BPG, and SIL-PG were initially dissolved in DMSO to obtain stock solutions of 1 mg/mL (for SIL) and 100 mg/mL (for BPG and SIL-PG), respectively. The solutions were further diluted in DMEM to achieve final concentrations of 0.9, 1.2, and 1.5 µg/mL for SIL and 300, 400, and 500 µg/mL for BPG and SIL-PG. The maximum DMSO concentration in culture medium (0.5%) was found non-cytotoxic during our experiments.

#### 4.4.2. 2D Cell Culture Protocol

This study was conducted using HaCaT keratinocytes (300493) delivered by Cytation, Eppelheim, Germany, in frozen vials and grown in their specific culture medium, namely DMEM supplemented with 10% FBS and 1% penicillin/streptomycin. The 2D cell line was maintained under standard conditions, representing 37 °C and 5% CO_2_, during the study. The cell line presented normal proliferation during the performed assays.

#### 4.4.3. MTT Cellular Viability Assessment

To evaluate the potential cytotoxicity of SIL, BPG, and SIL-PG in HaCaT cells, the viability was evaluated using the MTT assay. For this objective, 1 × 10^4^ cells/well were cultured in 96-well plates and incubated to reach the desired confluence (70%). After this step, HaCaT cells were treated for 24 h with SIL (0.9, 1.2, and 1.5 µg/mL), BPG, and SIL-PG (300, 400, and 500 µg/mL). To quantify cell viability after treatment, the medium was changed (100 µL/well), MTT reagent (10 µL/well) was added to each well, and the plates were incubated for a period of 3 h. Lastly, MTT solubilizing solution (100 µL/well) was added, and the plates were incubated for half an hour at room temperature. Absorbance was measured on Cytation 5 with readings performed at two wavelengths, 570 and 630 nm, respectively. Cell viability (%) was calculated as presented below:

(6) $$\mathrm{Cell}\;Viability\;(\%\;of\;control)\;=\;\;\left(\;\frac{Absorbance\;\;sample}{Absorbance\;Control}\right)\;\times \;100$$

#### 4.4.4. Bright-Field Assessment of Cell Morphology and Confluence

For the morphological analysis, HaCaT cells were seeded in 96-well plates and incubated until the desired confluence was reached (70%). After this stage, the cells were treated for 24 h with SIL (0.9, 1.2, and 1.5 µg/mL), BPG, and SIL-PG (300, 400, and 500 µg/mL), photographed, and analyzed using an IX73 microscope and the cellSens Dimensions software (version 1.8) from Olympus (Tokyo, Japan).

#### 4.4.5. Colony-Formation Assay

This assay was performed similarly to a protocol described in a previously published paper by Marcovici et al. [109]. The cells were seeded in 96-well plates at a low density of 100 cells/well. After attachment, the cells were exposed to SIL (0.9, 1.2, and 1.5 µg/mL), BPG, and SIL-PG (300, 400, and 500 µg/mL). After 24 h, the medium was replaced and regularly renewed throughout a 10-day incubation period. In the final stage, the cells were treated with 4% paraformaldehyde for 10 min at RT, washed with PBS, and stained with crystal violet (0.2% solution diluted in PBS) for 10 min. The dye was removed by washing the plates with distilled water several times. Cell lysis was performed using 1% SLS. The image acquisition of the formed HaCaT colonies was performed using Lionheart FX (BioTek Instruments Inc., Winooski, VT, USA). The absorbance was measured at 550 nm using a Cytation 5 device (BioTek Instruments Inc., Winooski, VT, USA). Image analysis was conducted with the Gen5 software. For colony-formation rate calculation, the ratio between the sample absorbance and the control absorbance was determined and multiplied by 100.

#### 4.4.6. Nuclear and Mitochondrial Staining

To assess the impact of SIL (0.9, 1.2, and 1.5 µg/mL), BPG and SIL-PG (300, 400, and 500 µg/mL) on the nuclei and mitochondria of HaCaT keratinocytes, the cells were seeded in 96-well plates and treated for 24 h after reaching optimal confluence (70%). Both mitochondrial and nuclear stainings were performed with MitoTracker™ Red CMXRos and Hoechst 33342, respectively. Briefly, the cells were first treated with MitoTracker™ Red CMXRos diluted in DMEM to a concentration of 300 nM. After 30 min with this staining solution, the cells were washed, treated with Hoechst 33,342 dye (diluted 1:2000 in PBS) for 5–10 min, washed three times again with PBS, and imaged using Lionheart FX at a magnification of 20×. The obtained images were analyzed using Gen5™ (version 3.14; BioTek Instruments Inc., Winooski, VT, USA).

#### 4.4.7. Generation and Treatment of HaCaT 3D Spheroid Culture

The scaffold-free development of HaCaT spheroids was performed by seeding the cells in 96-well, ultra-low attachment (ULA) plates at a density of 5000 cells/well and incubating them in standard conditions (37 °C, 5% CO_2_) for 72 h, similarly to the protocol reported by Vitacolonna et al. [110]. After this incubation period, HaCaT cells formed compact and well-defined spheroids with a diameter of around 200 µm. For the applied treatment, the culture medium was carefully removed, and the formed HaCaT spheroids were exposed to SIL (0.9, 1.2, and 1.5 μg/mL), BPG, and SIL-PG (300, 400, and 500 μg/mL) for 24 h.

#### 4.4.8. Spheroid Morphology and Viability Evaluation

After 24 h of treatment with SIL (0.9, 1.2, and 1.5 μg/mL), BPG, and SIL-PG (300, 400, and 500 µg/mL), HaCaT spheroids were imaged and measured using Lionheart FX and the Gen5 software. The viability was quantified using the Cell Titer-Glo^®^ 3D Luminescent Assay. In brief, fresh culture medium and Cell Titer-Glo^®^ 3D reagent (ratio of 1:1, *v*/*v*) were added to each well of the plates containing the spheroids, followed by orbital shaking of the plates for 20 min at room temperature to induce spheroid lysis. For luminescence reading on Cytation 5, the lysates were transferred to new white 96-well plates, and relative viability was determined as follows:

(7) $$Viability\;(\%\;of\;control)\;=\;\;\left(\;\frac{Luminescence\;sample}{Luminescence\;Control}\right)\;\times \;100$$

#### 4.4.9. The Skin Irritation Assay Using 3D EpiDerm^TM^ Reconstructed Human Tissues

To perform the irritation test, the EpiDerm^TM^ model (EPI-200-SIT) was procured from MatTek Corporation (Bratislava, Slovakia). Dulbecco’s phosphate-buffered saline (DPBS) served as the negative control (NC), while sodium dodecyl sulfate (SDS) 5% acted as the positive control (PC). The reagents required for the experiment, represented by media, solutions, and components of the MTT kit, were also sourced from the manufacturer. The irritant effect of the samples of interest, namely BPG and SIL-PG, was determined by performing a skin irritation test using EpiDermTM 3D reconstructed human tissue, based on the specific protocol [95]. Immediately after delivery, the tissues were cleaned of agarose, placed in new 6-well plates (0.9 mL of medium per well), and incubated for 1 h. Following this process, the medium was changed, and all inserts were incubated for 24 h. Consequently, the tissues were exposed for one hour to 30 μL of DPBS, 5% SDS, and samples of interest (BPG 500 µg/mL and SIL-PG µg/mL) that were applied to the apical region of the tissues. After treatment, the inserts were rinsed with DPBS and incubated for 24 h, followed by a new transfer to 6-well culture plates (with 0.9 mL of fresh medium/well) and another 18 h incubation period. At the end of these procedures, the inserts were placed in 24-well plates containing 0.3 mL of MTT solution in each well and incubated for 3 h at 37 °C. Then, the tissues were moved to a new 24-well plate, immersed in isopropanol (2 mL/well), and shaken for 2 h at RT. For quantification, absorbance was measured with Cytation 5 at 570 nm, and viability was calculated as presented by Pinzaru et al. in a previous study [31].

### 4.5. In Ovo Irritant Potential

#### 4.5.1. Chorioallantoic Membrane (CAM) Model Preparation

For this assay, fertilized hen eggs were used. Briefly, the hen eggs were cleaned and disinfected with 70% alcohol, then incubated under standard conditions (37.5 °C, 65% relative humidity) in an incubator specifically designed for this purpose. After 4 days, 5–10 mL of egg white was carefully extracted from each egg, and then a window was created in the upper part to allow a good view of the CAM, which was covered with medical adhesive tape.

#### 4.5.2. Evaluation of the Irritant Potential Using the HET-CAM Method

On day 10 of incubation, a sample volume of 600 µL was carefully applied to the chorioallantoic membrane, specifically the negative control (represented by H_2_O), the positive control (represented by SLS 1%—sodium lauryl sulfate), SIL 1.5 µg/mL, BPG 500 µg/mL, and SIL-PG 500 µg/mL. SIL, BPG, and SIL-PG test solutions were prepared by diluting the stock solutions (described in Section 4.4.1) in water. Each sample was thoroughly monitored for 5 min using a SteREO Discovery.V8 stereomicroscope (ZEISS, Jena, Germany) to detect any specific signs of irritation (lysis, hemorrhage, and coagulation), after which the irritation score (IS) was calculated using the formula presented in a previous work. From the results obtained from the calculation of IS, the sample can be classified as non-irritant if IS = 0–0.9, irritant if IS = 1–8.9, and severely irritant if IS = 9–21 [72].

### 4.6. Statistical Analysis

Statistical analysis was conducted throughout this study via the GraphPad Prism software, version 10.2.3 (GraphPad Software, San Diego, CA, USA, www.graphpad.com). The statistical differences between the treated groups and control (no treatment) or negative control (DPBS) were determined using one-way ANOVA and Dunnett’s multiple comparison tests. All the statistically significant results were marked with “*”, as follows: * *p* < 0.05; ** *p* < 0.01; *** *p* < 0.001; **** *p* < 0.0001.

## Figures and Tables

**Table 1:** Excipients used for the preparation of the blank proniosomal gel (BPG).

Component	Quantity/Volume
Sorbitan monostearate	180 mg
Cholesterol	30 mg
Soya lecithin	90 mg
Ethanol	0.3 mL
Distilled water	0.1 mL

**Table 2:** Regression and rheological parameters of Ostwald de Waele and Herschel–Bulkley models obtained for the tested proniosomal gels: blank proniosomal gel (BPG) and silibinin-loaded proniosomal gel (SIL-PG) (*n* = 3).

Rheological Model	BPG			SIL-PG		
	R^2^	K	n	R^2^	K	n
Ostwald de Waele (flow)	0.4998	135	0.075	0.3678	148.5	0.064
Ostwald de Waele (visc.)	0.9993	62.34	0.482	0.9991	65.98	0.488
Herschel–Bulkley (flow)	0.5429	2752	0.005	0.4041	2780	0.004
Herschel–Bulkley (visc.)	0.9998	95.97	0.277	0.9994	98.15	0.307

**Table 3:** Silibinin (SIL) solubility in different receptor media at 25 ± 2 °C. The experiment was conducted using three independent replicates (*n* = 3).

Receptor Medium	Solubility (mg/mL)
Phosphate-buffered saline pH 7.4 with 0.5% Brij^®^ 35	0.0307 ± 0.45
Phosphate-buffered saline pH 7.4 with 0.5% Brij^®^ S20	0.0263 ± 0.51
Phosphate-buffered saline pH 7.4 with 0.5% Eumulgin B 2 PH	0.0003 ± 0.82
Phosphate-buffered saline pH 7.4 with 1% Tween 20	0.0006 ± 0.34
Phosphate-buffered saline pH 7.4 with 1% Tween 80	0.0043 ± 0.72
Phosphate-buffered saline pH 7.4 with 30% ethanol	0.0301 ± 0.66
Phosphate-buffered saline pH 7.4 with 20% diethylene glycol monoethyl ether	0.5075 ± 0.43
Phosphate-buffered saline pH 7.4 with 30% diethylene glycol monoethyl ether	0.6079 ± 0.87
Phosphate-buffered saline pH 7.4 with 60% diethylene glycol monoethyl ether	5.8149 ± 1.26

**Table 4:** Results of in vitro silibinin (SIL) release and permeation from experimental proniosomal gel (*n* = 5).

Parameter	In Vitro SIL Release Through Synthetic Membrane	In Vitro SIL Permeation Through Pig Ear Skin
J_ss_ (μg/cm^2^/h)	34.30 ± 6.62	1.24 ± 0.04
K_P_ × 10^−6^ (cm/h)	114.33 ± 12.08	4.14 ± 0.12
t_L_ (h)	-	1.46 ± 0.61
k (μg/cm^2^/h^1/2^)	115.20 ± 13.88	7.33 ± 0.26

**Table 5:** Results of kinetic analysis of silibinin (SIL) in vitro permeability data, from the proniosomal gel through synthetic membrane and pig ear skin (*n* = 5).

Membrane Type/ Formulation Code	Zero Order		First Order		Higuchi		Korsmeyer–Peppas		
	K_0_ (μg/h)	R^2^	K_1_ (h^−1^)	R^2^	K_H_ (h^−0.5^)	R^2^	K_P_ (h^−n^)	n	R^2^
Synthetic membrane									
SIL-PG	7.92	0.9930	0.12	0.9832	18.63	0.9197	1.08	0.77	0.9993
Pig ear skin									
SIL-PG	0.23	0.9836	0.02	0.9818	0.81	0.7596	0.65	0.84	0.9592

*K*_0_: zero-order release constant; *K*_1_: first-order release constant; *K_H_*: Higuchi release constant; *K_P_*: Korsmeyer–Peppas release constant; *n*: diffusion coefficient in the Korsmeyer–Peppas model; R^2^: determination coefficient.

**Table 6:** Calculated irritation score (IS) for H_2_O, SLS 1%, SIL 1.5 μg/mL, BPG 500 μg/mL, and SIL-PG μg/mL using the HET-CAM assay. H_2_O was used as the negative control, and SLS at a 1% concentration represents the positive control. The experiment was conducted using three independent replicates (*n* = 3).

Sample	Calculated Irritation Score (IS)	Irritation Category
H_2_O	0.07 ± 0.02	Non-irritant
SLS 1%	19.94 ± 0.46	Severely irritant
SIL 1.5 μg/mL	0.07 ± 0.05	Non-irritant
BPG 500 μg/mL	0.45 ± 0.06	Non-irritant
SIL-PG 500 μg/mL	0.19 ± 0.05	Non-irritant

**Table 7:** Rheological models and corresponding equations used in the present study.

Rheological Model	Equation
Ostwald de Waele	$\tau =K\cdot {\dot{\gamma }}^{n}$ $\eta =K\cdot {\dot{\gamma }}^{n-1}$
Herschel–Bulkley	$\tau =\tau_{0}+K\cdot {\dot{\gamma }}^{n}$ $\eta =\eta_{0}+\tau_{0}/\dot{\gamma }$

## Data Availability

The data presented in this study are available on request from the corresponding author.
